# Diabetic Ketoacidosis and the Use of New Hypoglycemic Groups: Real-World Evidence Utilizing the Food and Drug Administration Adverse Event Reporting System

**DOI:** 10.3390/ph18020214

**Published:** 2025-02-05

**Authors:** Hilal A. Thaibah, Otilia J. F. Banji, David Banji, Thamir M. Alshammari

**Affiliations:** 1 Department of Clinical Practice, College of Pharmacy, Jazan University, Jazan 45142, Saudi Arabia; hthaibah@jazanu.edu.sa (H.A.T.); obanji@jazanu.edu.sa (O.J.F.B.); 2 Pharmacy Practice Research Unit, College of Pharmacy, Jazan University, Jazan 45142, Saudi Arabia; dbanji@jazanu.edu.sa; 3 Department of Pharmacology & Toxicology, College of Pharmacy, Jazan University, Jazan 45142, Saudi Arabia

**Keywords:** diabetic ketoacidosis (DKA), FDA adverse event reporting system (FAERS), hypoglycemic agents, real-world evidence (RWE), SGLT2 inhibitors

## Abstract

**Background**: Diabetic ketoacidosis (DKA), a life-threatening complication, can occur in individuals with type 2 diabetes during illness, stress, or medication use. This study examines DKA signals in type 2 diabetes, focusing on sodium–glucose cotransporter-2 (SGLT2) inhibitors, glucagon-like peptide-1 (GLP-1) receptor agonists, and dipeptidyl-peptidase-4 (DPP-4) inhibitors. **Methods:** DKA reports from Q1 2019 to Q3 2024 were retrieved from the FDA Adverse Event Reporting System (FAERS). Associations between primary exposure and outcomes were ascertained using four key metrics: Reporting Odds Ratio (ROR), Proportional Reporting Ratio (PRR), Empirical Bayes Geometric Mean (EBGM), and Information Component (IC). **Results:** SGLT2 inhibitors exhibited the higher DKA risk in 2019–2021 (ROR: 314.86 [95% CI 301.76–328.53], PRR of 245.69 [95% CI 235.47–256.36], IC of 6.90, and EBGM of 120), declining in 2022–2024. GLP-1 receptor agonists showed an ROR increase from 2.88 [95% CI 2.56–3.25] in 2019–2021 to 4.64 [95% CI 4.06–5.29] in 2022–2023, slightly declining to 3.95 [95% CI 3.27–4.74] in 2024. DPP-4 inhibitors exhibited a steady ROR rise from 6.81 [95% CI 5.52–8.40] in 2019–2021 to 8.57 [95% CI 6.24–11.76] in 2022–2023 and further to 11.02 [95% CI 6.71–18.10] in 2024. PRR, EBGM, and IC values followed similar trends. The age groups 41–60 and 61–91 years were the most affected, with hospitalization at its highest rate for DPP4-inhibitors in Q1–Q3 of 2024. Hospitalizations were also observed with GLP-1 receptor agonists and SGLT2 inhibitors. Life-threatening events and fatalities were also reported, with physicians contributing to most reports. **Conclusions:** DKA signals were observed for all three drug classes, particularly among elderly patients, highlighting the need for careful monitoring, especially during periods of illness or stress. However, the risk was higher in the SGLT2 inhibitor group than in the other groups.

## 1. Introduction

Several innovative drug classes have been developed for managing diabetes mellitus, offering additional benefits such as improved cardiorenal outcomes and weight reduction [1]. Among these, sodium–glucose cotransporter-2 (SGLT2) inhibitors are recommended by the American Diabetes Association (ADA) as the preferred treatment option for patients with type 2 diabetes mellitus (T2D) who are also affected by atherosclerotic cardiovascular disease, heart failure, or kidney disease [2].

In addition to SGLT2 inhibitors, dipeptidyl peptidase-4 (DPP-4) inhibitors, and Glucagon-Like Peptide-1 (GLP-1) receptor agonists have shown significant efficacy for T2D management. DPP-4 inhibitors prevent the degradation of GLP-1 in the gut, thereby enhancing insulin secretion and suppressing glucagon release [3,4]. In contrast, GLP-1 receptor agonists not only reduce hepatic glucose production and slow gastric emptying but also provide cardio-renal benefits. Furthermore, their metabolic actions have led to their widespread use in managing obesity [5].

Although these therapies have remarkable benefits, there are concerns regarding their association with diabetic ketoacidosis (DKA) [6,7,8,9]. DKA is a potentially life-threatening complication of diabetes that is characterized by high blood sugar levels, metabolic acidosis, and increased production of ketone bodies [10,11]. Most commonly, DKA is characterized by abdominal pain, nausea, and vomiting, leading to dehydration, in turn triggering postural hypotension and tachycardia. The resultant metabolic acidosis causes a form of deep breathing referred to as Kussmaul respiration [10,11]. Although DKA is observed in individuals with type 1 diabetes (T1D) owing to insulin deficiency, it can also occur in individuals with T2D, particularly during periods of illness, inadequate nutrition, dehydration, use of specific medications, or postoperative stress [12].

Regulatory agencies, including the United States Food and Drug Administration (U.S. FDA), have warned about the potential risk of DKA associated with SGLT2 inhibitors. Between March 2013 and June 2014, the FDA reported 20 cases of DKA, ketoacidosis, or ketosis, often presenting with mild hyperglycemia, leading to safety communication in 2015, alerting healthcare providers and patients about the risks and emphasizing the need for vigilance [13]. Consequently, ongoing assessments are crucial for ensuring patient safety [14]. FDA Adverse Event Reporting System (FAERS) data have revealed numerous DKA signals associated with SGLT2 inhibitors [7,15]. These reports covered a broad spectrum of patient characteristics, including age, body weight, and duration of medication use. While some DKA cases are linked to identifiable triggers, such as illness or reduced food intake, others occur without an apparent cause [16,17,18].

Similarly, GLP-1 receptor agonists have been linked to increased DKA risk [19]. In 2019, the United Kingdom (UK)’s Medicines and Healthcare Products Regulatory Agency (MHRA) issued advice regarding the potential risk of DKA in patients with T2D treated with a combination of a GLP-1 receptor agonist and insulin. This risk is evident when the insulin dose is reduced or stopped [20]. Dipeptidyl Peptidase-4 (DPP-4) inhibitors also carry this risk [21], with one study reporting the incidence rates of DKA at 6.0 and 4.3 per 1000 person-years for SGLT2 and DPP-4 inhibitors, respectively [22], while another study found rates of 4.9 events per 1000 person-years for SGLT2 inhibitors compared to 2.3 events per 1000 person-years for DPP-4 inhibitors [21].

FAERS is a data repository that collects real-world evidence of adverse events and medication errors from the United States and various international sources. Adverse event reports are submitted by healthcare professionals, manufacturers, patients, or consumers, making FAERS an essential resource for monitoring drug safety. Since FAERS collects adverse event reports from diverse populations, it becomes a vital source for identifying rare and serious adverse drug reactions, such as DKA. FAERS also contains a significant volume of reports on signals of newly approved drugs; hence, it is an ideal resource to evaluate emerging safety concerns associated with these medications. More importantly, the system is updated quarterly and is publicly accessible, providing timely insights into safety trends and rapid analysis of adverse event signals over time [23,24].

This study aims to comprehensively evaluate the association between using the newer classes of hypoglycemic agents—SGLT2 inhibitors, GLP-1 receptor agonists, and DPP-4 inhibitors—and their risk of developing DKA in patients with T2D. The study seeks to assess the magnitude of DKA signals linked to each drug class and identify patient characteristics that may influence DKA risk using real-world data from FAERS. Also, the study intends to identify the trends in reporting patterns before, during, and after the COVID-19 pandemic using pharmacovigilance metrics.

## 2. Results

**Figure 1:**
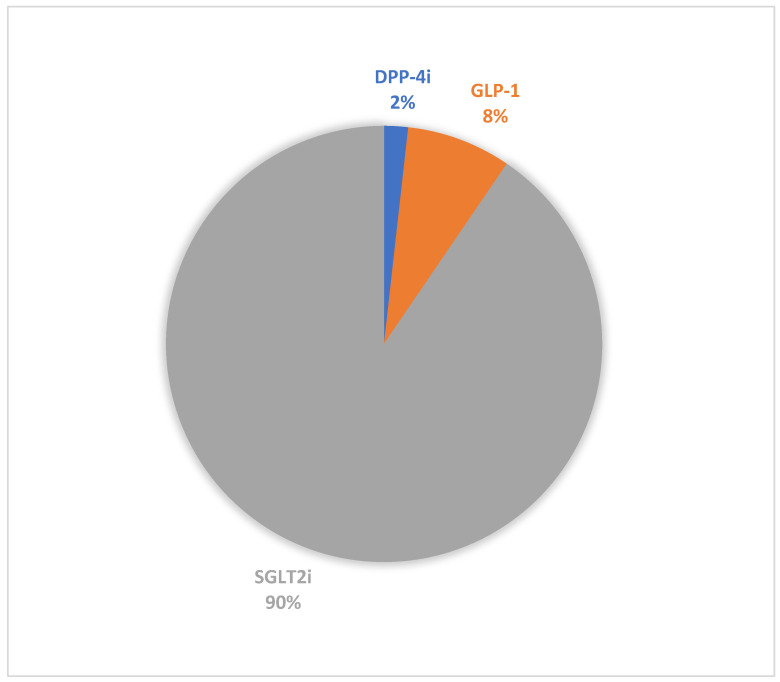
Proportions of DKA reports per medication class during the study period (2019–Q3 2024). DKA: diabetic ketoacidosis; DPP-4i: dipeptidyl peptidase-4 inhibitors; GLP-1: glucagon-like peptide-1 receptor agonists; SGLT2i: sodium–glucose cotransporter-2 inhibitors.

**Figure 2:**
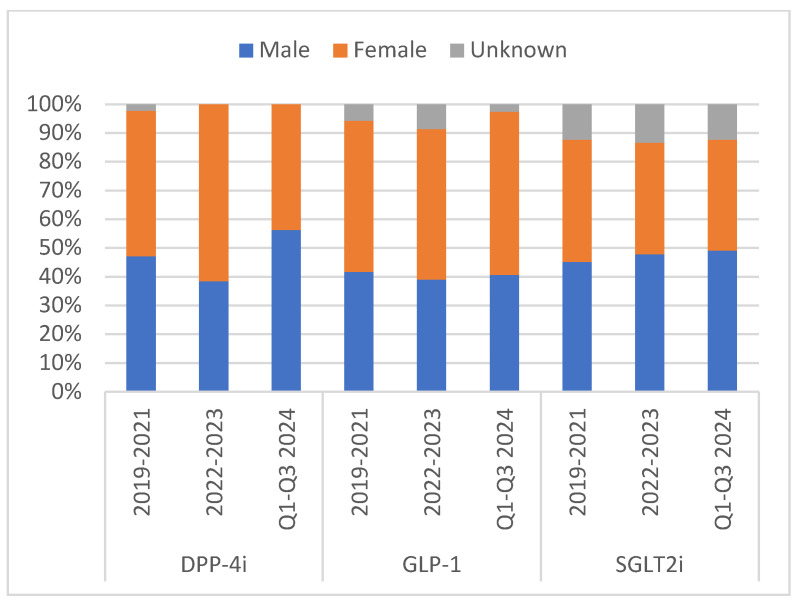
Proportions of patients’ gender from the DKA reports stratified by medication class and year. DKA: diabetic ketoacidosis; DPP-4i: dipeptidyl peptidase-4 inhibitors; GLP-1: glucagon-like peptide-1 receptor agonists; SGLT2i: sodium–glucose cotransporter-2 inhibitors; Unknown: missing data.

**Figure 3:**
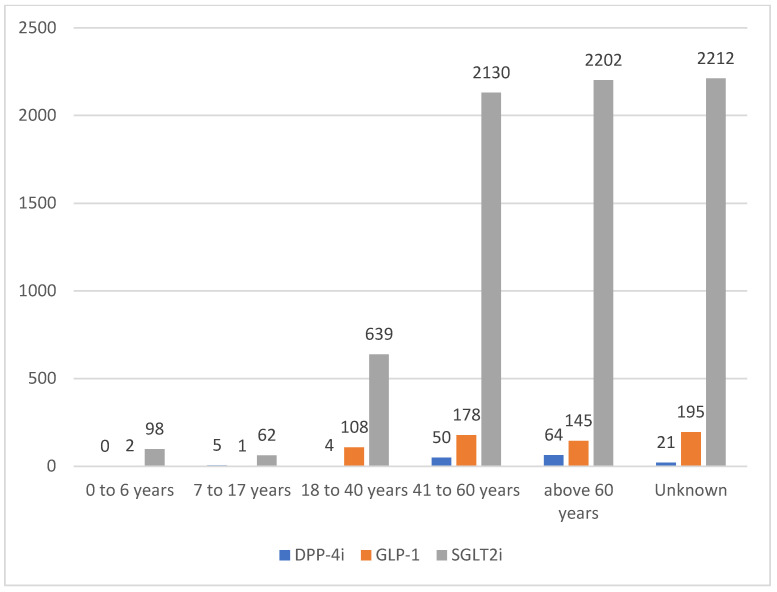
DKA reports stratified by patients’ age and medication class during the study period (2019–Q3 2024). DKA: diabetic ketoacidosis; DPP-4i: dipeptidyl peptidase-4 inhibitors; GLP-1: glucagon-like peptide-1 receptor agonists; SGLT2i: sodium–glucose cotransporter-2 inhibitors; Unknown: missing data.

The number of DKA cases reported across all medication classes during the study period was 8116. The reports consistently decreased over time. In Figure 1, SGLT2 inhibitors had the highest proportion of reports for DKA (*n* = 7343, 90.48%), followed by GLP-1 receptor agonists (*n* = 629, 7.75%) and DPP4 inhibitors (*n* = 144, 1.77%). The results showed interesting patterns in patient characteristics across all periods (Table 1). In Figure 2, the highest gender disparity was observed in the DPP-4 inhibitor class between 2022 and 2023, with approximately 23 points difference, with males comprising 38.64% compared to 61.54% females. However, in the SGLT2 inhibitor class of medications, reports from male patients consistently appeared to be higher than those from female patients. Additionally, 58.86% of the reports involved patients aged 41 years and above (*n* = 4769) across all medication classes during the study period (Figure 3). Physicians were the top reporters (35.59–66.67%) for all different hypoglycemic groups across all other periods in this study.

**Figure 4:**
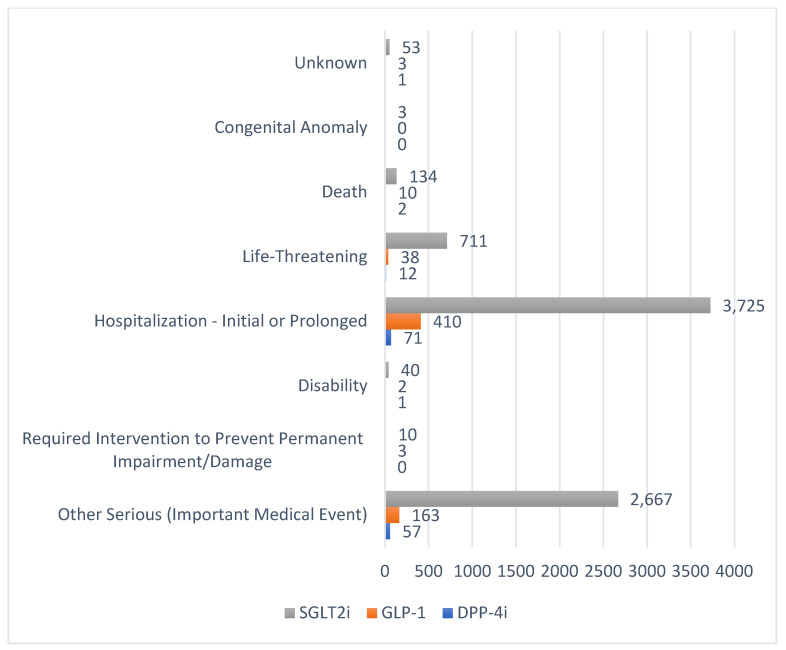
Outcomes of the reported DKA events per medication class during the study period (2019–Q3 2024). DKA: diabetic ketoacidosis; DPP-4i: dipeptidyl peptidase-4 inhibitors; GLP-1: glucagon-like peptide-1 receptor agonists; SGLT2i: sodium–glucose cotransporter-2 inhibitors; Unknown: missing data; outcome: the reported consequence of the DKA event and should be interpreted with caution as it does not entail causality.

Furthermore, most reports were from outside the U.S. (Table 1). The greatest disparity between the U.S. and non-U.S. reports was observed for DPP4 inhibitors in 2022–2023, while GLP-1 receptor agonists had the highest proportion of U.S.-originating reports in 2019–2021, amounting to over half the total reports (*n* = 140, 50.36%). Hospitalization or prolonged hospitalization was the most common outcome associated with the reported DKA events related to the hypoglycemic agents studied, followed by other serious or important medical events (Figure 4). Additionally, hospitalization was reported more with the GLP-1 receptor agonists by approximately 64.39% and 70.39% in 2019–2021 and 2022–2023, respectively. However, the risk of hospitalization or prolonged hospitalization was more reported (81%) with DPP-4 inhibitors in 2024 (Table 1).

There was a signal of DKA across all study periods using different hypoglycemic agents. In the period between 2019 and 2021, the signal was higher with the use of SGLT2 inhibitors (ROR 324.86, 95% confidence interval (CI) 301.76–328.53) compared with DPP-4 inhibitors and GLP-1 receptor agonists (ROR 6.81, 95% CI 5.52–8.40), (ROR 2.88, 95% CI 2.56–3.25), respectively). The risk was statistically significant along with other metrics, including PRR, EBGM, and IC (Table 2). Similar patterns are observed in 2022 and 2023. The PRR of the signal of DKA with the use of SLGT2 inhibitors was 114.61, 95% CI 107.92–121.73, while for DPP-4 inhibitors, it was 8.48, 95% CI 6.17–11.64, and for GLP-1 receptor agonists, it was 4.40, 95% CI 3.63–5.34 (Table 2). Finally, in 2024, there was also a DKA signal with hypoglycemic agents having the same patterns. The EBGM and IC were 38.46 and 5.26 for SGLT2 inhibitors, 8.30 and 3.05 for DPP-4 inhibitors, and 3.71 and 1.90 for GLP-1 receptor agonists, respectively (Table 2).

## 3. Discussion

Mining the FAERS database from Q1 2019 to Q3 2024 revealed potential adverse reactions associated with the three classes of antidiabetic medications. The data were analyzed across various periods to understand the likely patterns before, during, and after the coronavirus disease (COVID-19) pandemic. Therefore, the data were divided into three periods: pre-pandemic and pandemic years (2019 and 2021), post-pandemic years (2022–2023), and the available data for 2024 (i.e., January–September). Quantitatively validated methods such as disproportionality analyses have been employed to detect these signals. These methods, including PRR, ROR, IC, and EBGM, are widely utilized in pharmacovigilance systems such as FAERS [25,26,27]. Notably, these methods do not establish a causal relationship between drug exposure and adverse outcomes but are instrumental in identifying signals and trends in the association between signals and medication use [28].

Our analysis identified disparities in the risk factors for DKA associated with SGLT2 inhibitors, DPP-4 inhibitors, and GLP-1 receptor agonists. Between 2019 and 2021, SGLT2 inhibitors showed the highest positive risk signals for DKA, with an ROR of 314.86 (95% CI: 301.76–328.53), PRR of 245.69 (95% CI: 235.47–256.36), EBGM of 120, and IC of 6.90. However, a consistent decline in signals was observed in subsequent years. In 2022–2023, the ROR dropped to 126.96 (95% CI: 119.54–138.48) and the PRR to 114.61 (95% CI: 107.92–127.93). This decline continued for three quarters of 2024. Most signals were reported in the United States of America, likely because of the higher usage of SGLT2 inhibitors, which is 69.4% in the United States as against 16.7% in Europe and 11.7% in Asia [29]. Additionally, the surge in reporting, as observed in our data from 2019 to 2021, may be attributed to increased public awareness, possibly influenced by multiple FDA warnings [30,31].

The observed trend aligns with the documented risk of DKA associated with SGLT2 inhibitors [32,33]. Our findings are consistent with previous studies that analyzed individual case reports from the FAERS database. Fadini et al. reviewed FAERS data from Q1 2014 to Q3 2016 and identified 2397 cases of DKA among SGLT2 inhibitor users during this period [7]. Similarly, Vallabhajosyula et al. detected 979 DKA signals associated with SGLT2 inhibitors in 2183 drug-induced DKA cases [34]. In contrast, Zhou et al. detected high-intensity signals for euDKA, ketoacidosis, and DKA, with IC0.25 values of 7.65, 7.22, and 6.95, respectively, highlighting a significant association with these adverse events [35].

We did not observe sex-specific differences in the reported signals for SGLT2 inhibitors, which is consistent with other FAERS-based studies that reported similar rates of DKA signals among male and female users [34,36].

The prescription patterns of SGLT2 inhibitors vary across countries and are influenced by regulatory updates. In September 2023, revisions to the “warning and precaution sections” of the product labels for SGLT2 inhibitors highlighted the risk of DKA, particularly in patients with T1DM or those predisposed to ketoacidosis [13]. These updates and educational initiatives may have contributed to the subsequent reduction in the risk of DKA.

Although the decreasing trend in risk signals was positive, the risk of developing DKA associated with SGLT2 inhibitors persisted. This risk may be linked to their mechanism of action as they promote ketogenesis by lowering insulin requirements and increasing glucagon secretion, thereby altering the insulin-to-glucagon ratio. This imbalance enhanced hepatic ketogenesis and lipolysis. Additionally, glucosuria and decreased sodium reabsorption in the kidneys may increase ketone reabsorption. These factors collectively contribute to the accumulation of ketone bodies and the onset of DKA [17].

It is also important to emphasize that the available data does not confirm causality, which can only be established through extensive pharmacoepidemiological studies. Factors such as dehydration, surgical stress, and caloric restriction may independently increase the risk [17,37] and have not been reported. Given the substantial cardiovascular and renal benefits of SGLT2 inhibitors, it is crucial to weigh their potential risks carefully. Additionally, educating patients about the early signs of DKA, including nausea, abdominal discomfort, and mental confusion, can help manage the condition promptly. Advocating clinical guidelines recommending temporary discontinuation during acute illnesses or presurgical periods can mitigate these risks [38,39].

In contrast, the results of our study indicated that DPP-4 inhibitors produced significantly lower risk signals for DKA than SGLT2 inhibitors. The reduced risk of ketogenesis is most likely related to its mechanism of action, which enhances incretin activity to lower glucose levels without triggering ketogenesis [12,39]. However, a comparison of safety signals associated with the use of DPP-4 inhibitors across the years showed an upward trend in adverse event signals, showing an increase in ROR, PPR, and EBGM values, especially in the post-COVID-19 pandemic compared to previous years. However, it does not show a specific pattern of the pandemic impacting the risk of DKA in all three groups.

These results are intriguing, as the limited available evidence suggests the novelty of our findings. Nonetheless, the comparatively lower risk of DKA associated with DPP-4 inhibitors is consistent with the results of previous studies. For instance, one study reported incidence rates of 6.0 and 4.3 per 1000 person-years for SGLT2 and DPP-4 inhibitors, respectively [22]. Similarly, another study found rates of 4.9 events per 1000 person-years for SGLT2 inhibitors compared with 2.3 events per 1000 person-years for DPP-4 inhibitors [21].

Similarly, the data we analyzed showed that GLP-1 receptor agonists had a 1.5-fold increase in the risk of DKA from 2019–2021 to 2022–2023 and a 1.37-fold increase from 2019–2021 to 2024, indicating a gradual but noticeable increase in risk. Our findings align with the study of Yang et al., which also reported a significant association between GLP-1 receptor agonists and a risk of DKA (PRR 1.49, 95% CI: 1.24–1.79) [37]. The propensity for reduced ketogenesis may also be attributed to their mechanism of action, as they enhance glucose-dependent insulin secretion while simultaneously suppressing glucagon secretion. However, certain conditions, such as the off-label use of T1D or concurrent stress factors, may increase the risk of DKA [40,41,42]. However, GLP-1 receptor agonists are a favorable option because of their cardiovascular protection, weight loss benefits, and glycemic control [43].

Our study identified cases of hospitalization due to DKA-related symptoms, life-threatening conditions, and fatalities associated with all three classes of medications. Other researchers have reported similar findings by analyzing FAERS data on antidiabetic drugs. For instance, Fadini et al. have also documented a fatality rate for DKA linked to SGLT2 inhibitor use at 1.54% [7]. As the risk of congenital malformations associated with SGLT2 inhibitors was minimal in our study, this risk requires further investigation.

The analysis revealed that nearly 60% of the individuals aged 41–60 years exhibited a higher risk for DKA with the use of the three drug classes. The reporting pattern showed that physicians submitted most cases, followed by consumers, whereas pharmacists contributed less. These findings are consistent with those reported by Stottlemyer et al., who found that consumers accounted for 57.5% of FAERS submissions on antidiabetic medications, and physicians contributed 23% of the reports. In contrast, pharmacists have reported fewer reports [44].

There are limitations to this study that need to be considered. The study relied on data from spontaneous reports, a voluntary reporting system, which can result in over- or underreporting of adverse drug reactions. Individual case reports submitted to the FAERS database are in the form of self-reported information, which may omit essential details, such as comorbidities, polypharmacy, and disease severity, potentially leading to bias. The reports submitted do not adequately account for confounding factors like lifestyle choices, diet, or pre-existing health conditions that may lead to a risk of DKA. Although disproportionality measures are widely used for risk evaluation, they are not substitutes for clinical judgment. Also, these measures do not confirm causal relationships between drug exposure and adverse outcomes, necessitating further pharmacoepidemiological research for validation. Geographical disparities in reporting practices are seen plausibly because some countries have robust pharmacovigilance systems, making generalizability difficult. Also, reports submitted to FAERS lack denominator data, making risk estimation and quantification difficult. Despite these limitations, the continuous analysis of the FAERS data can help track changes in signal strength over time, indicating either remission or an increase in the risk associated with specific adverse events. In addition, to the best of our knowledge, this is the largest study comparing the three groups for almost six years.

## 4. Materials and Methods

### 4.1. Institutional Review Board Statement

Since this study utilized data from FAERS, a publicly available database of de-identified adverse event reports, no direct involvement of human or animal subjects occurred. Therefore, ethical approval from an Institutional Review Board (IRB) was not required. Additionally, the study complied with ethical standards for research involving publicly available data and followed all relevant guidelines for the responsible use of such data.

### 4.2. Study Design and Setting

This observational study used the FAERS database, which is freely available and can be accessed on the official U.S. FDA website (https://fis.fda.gov/extensions/FPD-QDE-FAERS/FPD-QDE-FAERS.html (accessed on 4 November 2024)) [45]. The datasets within the database were accessed and retrieved in November 2024. These databases are described in detail elsewhere [46]. Briefly, the reports in the databases are released quarterly and divided into seven primary datasets covering all variables in the U.S. FDA’s reporting form. These datasets were arranged into the following databases: Demographic and Administrative Information (DEMO), Drug Information (DRUG), Adverse Events (REAC), Patient Outcomes (OUTC), Report Sources (RPSR), Start and End Dates for Reported Drugs (THER), and Indications for Use (INDI). Furthermore, each dataset has classifications. For example, the reporter category is classified as physician, pharmacist, other health professional, consumer, and lawyer, while the outcome category was classified as death, life-threatening, hospitalization, disability, congenital anomaly, and required intervention to prevent permanent impairment or damage. Also, the reporter country is the country of the reporter of the last version of the report, either within or outside the United States. Therefore, the geographic area includes any countries that could send adverse drug reactions to FAERS. Age was defined as the numeric value of the patient’s age in the dataset at the event’s occurrence [45,46]. The primary identification number “primaryid” was used to link all the abovementioned datasets for this study.

The U.S. Food and Drug Administration (FDA) requires all pharmaceutical manufacturers to submit adverse drug reactions (ADRs) for their medications. Healthcare professionals and consumers/patients are encouraged to submit ADR reports to the FDA from or outside the U.S [24,47,48].

### 4.3. Data Collection and Management

This study was conducted between 2019 and 2024 to investigate the risk of DKA signals in the most recent hypoglycemic medication groups. Notably, during the study period, only three-quarters (i.e., January–September) of 2024 were available at FAERS. FAERS uses the Medical Dictionary for Regulatory Activities (MedDRA) coding system to process the adverse events (AEs) using preferred terms (PT). Therefore, the PT was used to determine the outcome of interest, which were diabetic ketoacidosis (DKA) and euglycemic DKA that we have used in this study. Multiple terms were used to identify all the possible reports related to DKA. These terms include “diabetes”, “diabetic”, “keto”, and “ketoacidosis.” Additionally, the research team performed further checks by tabulating the outputs to ensure all included reports were related to DKA only.

The exposures of interest were the reports that included one of the main hypoglycemic medication groups: DPP-4 inhibitors (sitagliptin, saxagliptin, linagliptin, and alogliptin), SGLT2 inhibitors (canagliflozin, dapagliflozin, and empagliflozin), and GLP-1 receptor agonists (exenatide, liraglutide, dulaglutide, semaglutide, and albiglutide). The reports of drugs of interest were retrieved using both the “drugname” and “prod_ai” variables in the databases.

Combinations of hypoglycemic medications from the different groups used in this study were excluded to obtain more accurate results. In addition, since there are multiple options for the association between the outcome of interest and the exposure of interests, such as primary suspected (PS), secondary suspected (SS), and concomitant medication, only PS was included in this study to minimize bias. The “primaryid” along with the event date (event_dt) and also the “pt” were used to ensure that there were no duplicates in reports.

### 4.4. Statistical Analysis

A pharmacovigilance case/non-case disproportionate method was used to measure the risk of DKA development with SGLT2 inhibitors, DPP-4 inhibitors, and GLP-1 receptor agonists. The cases were the reports of DKA linked to each exposure group of interests, whereas the “no-case” was all other AE reports linked to each exposure group of interests other than DKA.

Bayesian and traditional statistical analysis methods were used to investigate the potential safety concerns related to drug combinations and adverse events. A 2 × 2 contingency table is used to mine the data for the four metrics. The table consists of components (cells): a, b, c, and d. “A” represents the number of reports of cases (outcome of interest) for the studied medications, and “b” is the number of reports of non-cases (no outcome of interest adverse events) for those medications. In addition, “c” represents the number of reported cases for other medications, and “d” represents the number of reports of non-cases for all other medications (Table 3) [46].

We used this dual-method approach to enhance the analysis’s rigor and avoid incorrect findings. These methods include computing four metrics: empirical Bayes geometric mean (EBGM), information component (IC), the reporting odds ratio (ROR), and proportional reporting ratio (PRR) [24]. A signal above one for ROR indicated a potential safety signal. In the case of PRR, a safety signal was considered significant at values of two and above. An EBGM above the threshold of two or more is also indicative of a safety signal regarding exposure associated with DKA [24,46]. The threshold value of the IC metric for any value above zero indicates a safety signal (Table 4) [24,49].

Significant signals had to fulfill the criteria across all four metrics to ensure the reliability of the findings. This method guarantees that only dependable and consistent signals are identified, thereby reducing the likelihood of incorrect associations. All statistical analyses were conducted using the statistical software R (version 4.2.2) and R Studio (Version 2024.04.2+764).

## 5. Conclusions

Analysis of DKA signals across hypoglycemic agents revealed distinct risk profiles among the drug classes, with SGLT2 inhibitors demonstrating the highest risk. In comparison, GLP-1 receptor agonists and DPP-4 inhibitors exhibit a lower risk of DKA, likely due to their mechanisms of action, which do not directly promote ketogenesis. However, given the severity of DKA, clinicians should consider this risk across all groups when tailoring the therapeutic plan for each patient. Regular assessment of patient hydration status, nutritional intake, or comorbid conditions is vital to prevent complications. Most importantly, monitoring ketone levels, especially during acute illness, physical or emotional stress, reduced food intake, or before surgery, can be crucial in detecting the early signs of DKA. Additionally, risk assessment tools that factor in age, renal function, and medication use can significantly assist clinicians in identifying high-risk patients. The findings highlight the importance of pharmacovigilance and adherence to clinical practice guidelines to minimize risks and improve patient safety.

## Figures and Tables

**Table 1:** Frequency of DKA reports stratified by medication class, years, and reported characteristics.

Medication Class	DPP-4i *						GLP-1 **						SGLT2i ^§^					
Duration	2019–2021		2022–2023		Q1–Q3 2024		2019–2021		2022–2023		Q1–Q3 2024		2019–2021		2022–2023		Q1–Q3 2024	
***Characteristics***	*n*	(%)	*n*	(%)	*n*	(%)	*n*	(%)	*n*	(%)	*n*	(%)	*n*	(%)	*n*	(%)	*n*	(%)
***Patient’s Age (years)***																		
0–6	-	-	-	-	-	-	1	0.36	-	-	1	0.85	58	1.18	32	1.75	8	1.35
7–17	4	4.49	1	2.56	-	-	-	-	-	-	1	0.85	35	0.71	23	1.26	4	0.68
18–40	2	2.25	1	2.56	1	6.25	47	16.9	38	16.3	23	19.5	506	10.3	115	6.29	18	3.05
41–60	32	36	14	35.9	4	25	77	27.7	67	28.8	34	28.8	1548	31.4	432	23.7	150	25.4
>60	36	40.5	20	51.3	8	50	61	21.9	58	24.9	26	22	1354	27.5	620	33.9	228	38.6
Unknown	15	16.9	3	7.69	3	18.8	92	33.1	70	30	33	28	1424	28.9	605	33.1	183	31
***Reporter’s Occupation***																		
Physician	46	51.7	26	66.7	8	50	115	41.4	83	35.6	42	35.6	2567	52.1	1080	59.1	327	55.3
Pharmacist	4	4.49	2	5.13	5	31.3	18	6.47	21	9.01	11	9.32	651	13.2	271	14.8	89	15.1
Other Health Professional	16	18	9	23.1	3	18.8	43	15.5	52	22.3	25	21.2	969	19.7	339	18.6	112	19
Lawyer	2	2.25	-	-	-	-	-	-	-	-	-	-	1	0.02	1	0.05	-	-
Consumer	19	21.4	2	5.13	-	-	97	34.9	77	33.1	40	33.9	410	8.32	126	6.9	60	10.2
Unknown	2	2.25	-	-	-	-	5	1.8	-	-	-	-	327	6.64	10	0.55	3	0.51
***Reporter’s Country***																		
United States	30	33.7	3	7.69	4	25	140	50.4	74	31.8	38	32.2	1106	22.5	674	36.9	255	43.2
Outside the United States	57	64	36	92.3	12	75	137	49.3	159	68.2	80	67.8	3814	77.4	1153	63.1	336	56.9
Unknown	2	2.25	-	-	-	-	1	0.36	-	-	-	-	5	0.1	-	-	-	-
***Total DKA Reports***	**89**		**39**		**16**		**278**		**233**		**118**		**4925**		**1827**		**591**	

Abbreviations: 1 DKA: diabetic ketoacidosis; DPP-4i: dipeptidyl peptidase-4 inhibitors; GLP-1: glucagon-like peptide-1 receptor agonists; SGLT2i: sodium–glucose cotransporter-2 inhibitors; Unknown: missing data. * DPP-4i included sitagliptin, saxagliptin, linagliptin, and alogliptin, ** GLP-1 included exenatide, liraglutide, dulaglutide, semaglutide, and albiglutide, and ^§^ SGLT2i included sitagliptin, saxagliptin, linagliptin, and alogliptin.

**Table 2:** Risk of DKA and the use of hypoglycemic agents during study periods.

Medication Class	Duration	DKA Reports	Reported ADEs	ROR	PRR	EBGM	IC
				(95% CI)	(95% CI)		
DPP-4i	2019–2021	89	7333	6.81	6.74	6.48	2.69
				(5.52–8.40)	(5.46–8.31)		
	2022–2023	39	3373	8.57	8.48	7.69	2.94
				(6.24–11.76)	(6.17–11.64)		
	Q1–Q3 2024	16	925	11.02	10.85	8.3	3.05
				(6.71–18.10)	(6.60–17.82)		
GLP-1	2019–2021	278	54,286	2.88	2.87	2.81	1.5
				(2.56–3.25)	(2.55–3.24)		
	2022–2023	233	38,192	4.64	4.4	4.41	2.14
				(4.06–5.29)	(3.63–5.34)		
	Q1–Q3 2024	118	19,563	3.95	3.93	3.71	1.9
				(3.27–4.76)	(3.26–4.74)		
SGLT2i	2019–2021	4925	22,348	314.86	245.69	120	6.9
				(301.76–328.53)	(235.47–256.36)		
	2022–2023	1827	18,641	126.96	114.61	70	6.13
				(119.54–138.48)	(107.92–121.73)		
	Q1–Q3 2024	591	9258	60.48	56.68	38.46	5.26
				(54.78–66.76)	(51.34–62.58)		

DKA: diabetic ketoacidosis; ADEs: adverse drug event; DPP-4i: dipeptidyl peptidase-4 inhibitors; GLP-1: glucagon-like peptide-1 receptor agonists; SGLT2i: sodium–glucose cotransporter-2 inhibitors; ROR: reporting odds ratio; PRR: proportional reporting ratio; EBGM: empirical Bayes geometric mean; IC: information component.

**Table 3:** Contingency table for disproportionality analyses.

	Adverse Drug Reaction of Interest	Other Adverse Drug Reactions
**Drug of Interest**	a	b
**All Other Drugs in the Database**	c	d

**Table 4:** Formulas of disproportionality metrics and their criteria.

Metric	Formula	Criteria
**ROR, 95% CI**	$\frac{a/b}{c/d}$ $95\%\mathrm{CI}=e^{In\left(ROR\right)\pm 1.96\sqrt{\frac{1}{a}+\frac{1}{b}+\frac{1}{c}+\frac{1}{d}}}$	95% CI > 1, *n* ≥ 2
**PRR, 95% CI**	$\frac{a/(a+b)}{c/(c+d)}$ $95\%\mathrm{CI}=e^{In\left(PRR\right)\pm 1.96\sqrt{\frac{1}{a}+\frac{1}{b}+\frac{1}{c}+\frac{1}{d}}}$	PRR ≥ 2, chi-squared ≥ 4, *n* ≥ 3
**EBGM**	$\frac{a(a+b+c+d)}{\left(a+c)(a+b\right)}$	EBGM 05 ≥ 2
**IC**	${\mathrm{Log}}_{2}\;\frac{a(a+b+c+d)}{\left(a+c)(a+b\right)}$	Lower limit of 95% CI ≥ 0

CI: confidence interval; EBGM: empirical Bayes geometric mean; IC: information component; *n*: number of reports; PRR: proportional reporting ratio; ROR: reporting odds ratio.

## Data Availability

The data are available upon request from the corresponding author.
